# GOLEM: A tool for visualizing the distribution of Gene regulatOry eLEMents within the plant promoters with a focus on male gametophyte

**DOI:** 10.1111/tpj.70037

**Published:** 2025-03-02

**Authors:** Lukáš Nevosád, Božena Klodová, Jiří Rudolf, Tomáš Raček, Tereza Přerovská, Alžbeta Kusová, Radka Svobodová, David Honys, Petra Procházková Schrumpfová

**Affiliations:** ^1^ National Centre for Biomolecular Research, Faculty of Science Masaryk University Kotlářská 2 611 37 Brno Czech Republic; ^2^ Laboratory of Pollen Biology Institute of Experimental Botany of the Czech Academy of Sciences Rozvojová 263 165 02 Prague Czech Republic; ^3^ Central European Institute of Technology Masaryk University Kamenice 5 625 00 Brno Czech Republic

**Keywords:** gametophyte, Gene regulatOry eLEMents, GOLEM, motif localization, plant genes, promoter elements, technical advance, TSS

## Abstract

Gene expression regulation during tissue development is extremely complex. A key mechanism of gene regulation is the recognition of regulatory motifs, also known as cis‐regulatory elements (CREs), by various proteins in gene promoter regions. Localization of these motifs near the transcription start site (TSS) or translation start site (ATG) is crucial for transcription initiation and rate. Transcription levels of individual genes, regulated by these motifs, can vary significantly across tissues and developmental stages, especially in processes like sexual reproduction. However, the precise localization and visualization of these motifs in relation to gene expression in specific tissues can be challenging. Here, we introduce a freely available tool called GOLEM (Gene regulatOry eLEMents; https://golem.ncbr.muni.cz), which enables users to precisely locate any motif of interest with respect to TSS or ATG within the relevant plant genomes across the plant Tree of Life (*Chara*, *Marchantia*, *Physcomitrium*, *Azolla*, *Ceratopteris*, *Amborella*, *Oryza*, *Zea*, *Solanum* and *Arabidopsis*). The visualization of the motifs is performed with respect to the transcript levels of particular genes in leaves and male reproductive tissues and can be compared with genome‐wide distribution regardless of the transcription level. Additionally, genes with specific CREs at defined positions and high expression in selected tissues can be exported for further analysis. GOLEM's functionality is illustrated by its application to conserved motifs (e.g. TATA‐box, ABRE, I‐box, and TC‐element), hormone‐responsive elements (GCC‐box, ARR10_binding motif), as well as to male gametophyte‐related motifs (e.g., LAT52, MEF2, and DOF_core).

## Significance Statement

Precise localization and visualization of gene regulatory motifs in gene promoters, in relation to gene transcription in specific tissues, can be challenging, especially for researchers without advanced bioinformatics expertise. GOLEM (Gene regulatOry eLEMents) is a freely available tool that enables users to precisely locate any motif of interest with respect to the TSS or ATG within plant genomes across evolution, with respect to the transcript levels of specific genes in leaves and male reproductive tissues.

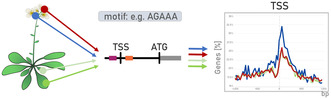

## INTRODUCTION

The regulation of gene expression is a dynamic process that requires a tightly orchestrated control mechanism. This regulation is crucial not only for maintaining proper cellular function but also for precise regulation of gene transcription and control of cell differentiation into specific tissues and organs. Cis‐regulatory elements (CREs) are short DNA sequence motifs that act as molecular switches activating or repressing gene expression (Galli et al., 2020; Schmitz et al., 2022). In order to do that, CREs serve as binding sites for various regulatory proteins, including transcription factors (TFs) (Preissl et al., 2023).

When performing CRE localization within the plant or animal genomes, FIMO (Find Individual Motif Occurrences) or CentriMoLocal (Motif Enrichment Analysis) that are part of the MEME suite (Bailey et al., 2015; Grant et al., 2011) are popular tools. However, users must consider certain limitations, such as uploading input already pre‐processed data in specific formats and understanding proper parameter settings. Moreover, analyzing DNA sequences from promoter regions relevant to genes with high transcription in specific tissues, based on RNA‐seq data, can be challenging for those lacking bioinformatics expertise, even when using the web interface with preconfigured settings. Additionally, inconsistencies in gene nomenclature systems in many plant species (Blaby et al., 2014; McCouch, 2008; Pan et al., 2023; Rensing et al., 2020), compared to well‐established model organisms (e.g., thale cress, human), can pose obstacles for the automatization of these procedures.

Transcriptomics has gained significant popularity in recent years, as it can provide detailed insights into gene expression dynamics across different tissues, developmental stages, or experimental conditions (Tyagi et al., 2022). Transcriptome sequencing (RNA‐Seq) is an important method for investigating gene regulation. While most transcriptome analyses have traditionally focused on easily accessible plant materials such as leaves or seedlings, there is a growing trend toward exploring transcriptomes from intricate and deeply embedded tissues, including sperm cells and various pollen developmental stages (Julca et al., 2021; Klodová et al., 2023). These innovations, together with advances in bioinformatics, are facilitating breakthroughs in understanding the regulation of plant reproduction. By determining overrepresented CREs in the promoters of differentially expressed genes, the candidate tissue‐specific transcriptional regulators can be identified (Shi et al., 2021).

The first identified eukaryotic CRE within the gene promoters, owing to its predictable locations surrounding gene transcription start sites (TSS), was TATA‐box (TATAWA) (Feng et al., 2016; Lifton et al., 1978; Suzuki et al., 2001). The TATA‐box has been conserved throughout the evolution of eukaryotes and is usually located 25–35 base pairs (bp) upstream of TSS (McKnight & Kingsbury, 1982). Even though the TATA‐box is a common CRE, it is not a general feature of all promoters. Only a fraction of eukaryotic genes actually harbor a TATA‐box: 20–46% of promoters in yeast (Basehoar et al., 2004; Yang et al., 2007) or less than 10% of genes in human (Carninci et al., 2006; Shi & Zhou, 2006). In plants, less than 39% of thale cress (*Arabidopsis thaliana*) promoters contain a TATA‐box or a TATA‐variants (Bernard et al., 2010; Savinkova et al., 2023), whereas approximately 19% of rice (*Oryza sativa*) genes possess the TATA‐box (Civán & Svec, 2009). In recent years, it became apparent that there are no universal promoter elements across species, and some promoter elements are involved in enhancer‐promoter specificity as well as specific biological networks. However, precise regulatory sequences at precise locations are essential for promoter function (Pavlu et al., 2024; Vo Ngoc et al., 2017).

Specific CREs can contribute to the expression of particular target genes in response to hormonal stimuli or stress (Vo Ngoc et al., 2017). For instance, the core sequence GATY is recognized by type‐B Arabidopsis Response Regulators (ARR10), proteins that mediate the cytokinin primary response (Hosoda et al., 2002; Šmeringai et al., 2023; Xie et al., 2018). The ethylene‐responsive element GCC‐box (core sequence GCCGCC) is present in many ethylene‐inducible pathogenesis‐related genes (Eyal et al., 1993; Hao et al., 1998; Ohme‐Takagi & Shinshi, 1990), whereas the DRE/CRT (CCGAC) element is an important element present in genes connected to abiotic stress, especially water unavalibility (Agarwal et al., 2017). Concerning organ development, the I‐box is involved in light‐regulated and/or leaf‐specific gene expression of photosynthetic genes (Castresana et al., 1987; Gidoni et al., 1989; Manzara et al., 1991). The I‐box motifs (GATAAG) were found in most of the genes crucial for plant photosynthesis (Castresana et al., 1987; Gidoni et al., 1989).

Plant sexual reproduction possesses an extraordinary ability to establish new cell fates throughout their life cycle, in contrast to most animals that define all cell lineages during embryogenesis (She & Baroux, 2014). Sexual reproduction was introduced after the origin of meiosis (Meissner, 2021) and the life cycle, in which diploid sporophytes alternate with the haploid gametophyte in land plants (Williams & Reese, 2019). The key mediators of developmental and organismal phenotypes are CREs, which can orchestrate precise timing and magnitude of gene transcription, especially during the development of male or female gametophyte tissues (Marand et al., 2023). Several CREs involved in the regulation of key genes required for the differentiation of male germline, active in sperm cells and pollen vegetative cells, have already been identified: MEF2‐type CArG‐box (CTA(A/T)_4_TAG; Verelst et al., 2007); LAT52 pollen‐specific motif in tomato (AGAAA; Bate & Twell, 1998); DOF core motif (AAAG; Li et al., 2014; Yanagisawa, 2002); and many others (Hoffmann et al., 2017; Li et al., 2014; Peters et al., 2017; Sharma et al., 2011). However, the precise distribution of these motifs within promoters, particularly their proximity to TSS or translation start site (ATG) and their prevalence in the promoters of genes exhibiting higher transcription levels in specific tissues related to plant reproduction, remains unclear.

Here we present a user‐friendly online software, GOLEM (Gene regulatOry eLEMents) https://golem.ncbr.muni.cz, which allows browsing various tissues such as sporophyte (leaves) or male gametophyte developmental tissues (antheridia, pollen stages, sperm cells) across the selected plant genomes within the plant Tree of Life (streptophyte algae, mosses, ferns, basal angiosperms, monocots, and dicots). Our software enables us to investigate the precise localization and distribution of any CREs of interest in gene promoters, in proximity to the TSS and ATG. The set of investigated genes can be specified by the level of gene expression in specific tissues based on transcriptomic data. Furthermore, tracking the genome‐wide distribution across exemplified genomes, regardless of the transcription level, may aid in tracking the evolution of regulatory motifs across the plant Tree of Life. Finally, a set of genes with only specific CREs at defined positions showing high expression only in the tissue of interest can be exported for further analysis, including for instance, protein functional enrichment analysis. We demonstrate the utilization of the GOLEM program not only on motifs associated with male gametophyte development, such as LAT52, MEF2, and DOF_core, but also on hormone‐responsive elements (GCC‐box, ARR10_core) or conserved motifs such as the TATA‐box, ABRE, TC‐element, I‐box and DRE/CRT element.

## RESULTS AND DISCUSSION

### Gene expression dynamics during the male gametophyte development

**Figure 1:**
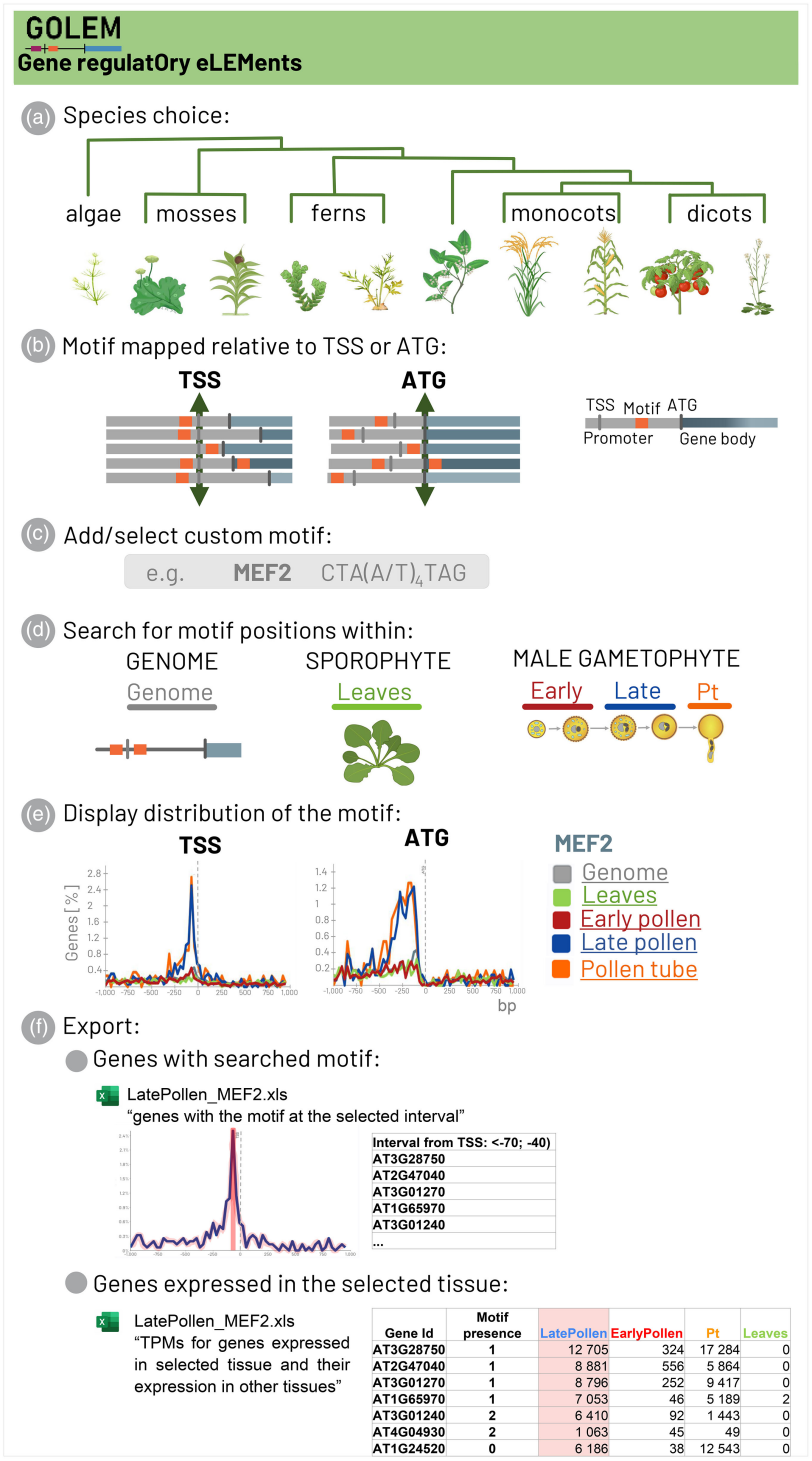
Illustrative overview of the workflow of the GOLEM software. (a) The plant species across the plant Tree of Life is chosen. (b) Region in the vicinity of the transcription start site (TSS) or translation start site (ATG) is specified. (c) Motifs of interest are defined. (d) The promoters of the genes showing expression in selected tissue (sporophyte, male gametophyte), together with an analysis of genome‐wide distribution regardless of transcription, are chosen for the analysis. (e) The exemplified MEF2‐type CArG‐box motif shows distribution upstream of TSS, with higher prevalence in the promoters of genes transcribed during late pollen development. (f) The accession numbers (gene ID) of genes with certain motifs within a defined region and tissue, or genes expressed in selected tissues, may be exported in XLSX format tables.

User‐friendly online software GOLEM allows browsing various tissues such as leaves, leaflets, or tissues associated with plant sexual reproduction (antheridia, pollen stages, sperm cells) across the selected plant genomes and investigates the precise localization and distribution of any CREs of interest in gene promoters, in proximity to the TSS and ATG. The set of investigated genes, in each tissue or in individual pollen developmental stages, can be specified by the level of gene expression in specific tissues based on transcriptomic data and calculated values of TPM (Figure 1). However, plant sexual reproduction is a complex process involving specialized structures at several stages that can significantly differ in the level of their transcription (Bokvaj et al., 2015; Hafidh et al., 2016).

Land plants evolved from streptophyte algae, where the haploid gametophyte generation often dominates the life cycle over the diploid phase. In *Chara braunii*, one of the streptophyte algae with the most complex body plans, male gametes (sperm cells) are released from male gametangia (antheridia) (Nishiyama et al., 2018). Similarly, in bryophytes such as the liverwort (*Marchantia polymorpha*) and mosses (*Physcomitrium patens*), the haploid gametophyte generation is the dominant phase of the life cycle, and sperm cells are released from male gametangia (antheridia) (Kohchi et al., 2021; Rensing et al., 2020). In contrast, ferns such as *Azolla filiculoides* and *Ceratopteris richardii* exhibit a longer‐lived diploid sporophytic generation compared to the haploid gametophytic generation. Nevertheless, sperm cells in ferns are also released from antheridia, which are present on sexually differentiated gametophytes (Atallah & Banks, 2015; Sebastian et al., 2021).

Flowering plants have highly reduced male and female gametophytes. During the early stages of male gametophyte (pollen) development, the haploid uninucleate microspore (UNM) divides asymmetrically to form bicellular pollen (BCP), which is comprised of a large vegetative cell and a small generative cell in a unique “cell‐within‐a‐cell” structure. In approximately 30% of angiosperms, including *A. thaliana*, *O. sativa*, and *Zea mays* the generative cell divides again to form tricellular pollen (TCP; reviewed in Hafidh and Honys, 2021) so that the mature pollen grain (MPG) is tricellular, composed of the vegetative cell and two sperm cells. After reaching the stigma, the growing pollen tube (PT) is guided to the female gametophyte (ovules) to deliver the sperm cells. In 70% of species, including *Solanum lycopersicum* and basal angiosperms as *Amborella trichopoda* (Williams et al., 2014), the MPG is bicellular and becomes tricellular after the MPG reaches the papillary cells of the stigma, where it is rehydrated and activated (reviewed in Hafidh et al., 2016; Johnson et al., 2019).

GOLEM is based on comparing the expression of individual genes across various developmental stages and tissue samples based on TPM. In many angiosperms, including *A. thaliana* or *Nicotiana tabacum*, a substantial reduction in the number of expressed genes and significant changes during the transition from early pollen stages (UNM, BCP) to late pollen stages (TCP, MPG) were reported (Hafidh et al., 2018; Klodová et al., 2023). Due to the significant differences in gene expression between the stages, TPMs between various stages cannot be compared directly (Zhao et al., 2020). To overcome this, the sequential values of a series of TPM numbers are pooled and compared, as this enables comparison across multiple samples with varying numbers of input values. The genes with the highest transcription in each stage can be set as a percentile (e.g., the 90^th^ percentile comprises the genes whose transcripts represent 90% of all transcripts transcribed from the total number of protein‐coding genes) or as a certain number of the genes. The number of genes in the chosen percentile, from the total number of the validated genes included in the analysis, can be tracked in the GOLEM outputs. The results can be further displayed as percentages of the genes with certain motifs; however, the exact counts of the motifs can be tracked alongside (Figure S1).

Our analysis confirmed the reduction in the number of expressed genes at late pollen developmental stages described previously in Honys and Twell (2003, 2004) and Klodová et al. (2023). In early pollen stages, leaves and seedlings of *A. thaliana* the genes composing the 90^th^ percentile, represent 27, 24 and 30% of the total protein‐coding genes, respectively. In late pollen stages, sperm cells and PT, those genes represent 7, 3 and 5%, respectively (Figure S2a). On the other hand, in the bryophyte *M. polymorpha*, the genes whose transcripts comprise the 90^th^ percentile show more similar levels in antheridia, sperm cells, and thallus, 25, 30 and 29%, respectively (Figure S2b).

### Positional distribution of peaks reveals preferential localization of the searched motifs to the TSS or ATG

The GOLEM software aligns all genes relative to the TSS or ATG and conducts a comprehensive analysis of the CREs in their proximal regions (Figure 1b). When comparing the results from TSS and ATG‐based analyses, it is crucial to consider the variations in the length of the 5′ UTR. The median length of the 5′ untranslated regions (5′UTRs), i.e., the region between the TSS and ATG, is not uniform across the plant species. The median length of 5′UTR is 454 bp in *M. polymorpha* (Bowman et al., 2017); 477 bp in *P. patens* (Zhang et al., 2021); 111 bp in *O. sativa* (Srivastava et al., 2018; Zhang et al., 2021); 179 bp in *Z. mays* (Zhang et al., 2021); 214 bp in *S. lycopersicum* (Zhang et al., 2021); and 184 bp in *A. thaliana* (Zhang et al., 2021). However, genes with very short (1–50 bp) or very long (>2000 bp) 5′UTRs were also detected (Hafidh et al., 2018; Klodová et al., 2023). Due to the varying lengths of the 5′UTR, it is possible to determine the positional distribution of the peak near the TSS or ATG (whether it is sharp, narrow, or bell‐shaped) (Yu et al., 2016). It enables determining whether the motif of interest is preferentially localized near the TSS or ATG.

The positional distribution and revelation of the preferential motif localization can be exemplified by the TC‐elements, dehydration‐responsive element/C‐repeat (DRE/CRT) and ABA‐responsive cis‐element‐coupling element 1 (ABRE motif) (Figures S1c,g and S3). TC‐element (TC_(n)_, TTC_(n)_) is a motif described in *A. thaliana* and *O. sativa* promoters but not in *Homo sapiens* or *Mus musculus*. TC‐element is preferentially present in the promoters of genes involved in protein metabolism (Bernard et al., 2010). The authors showed the peak is centered −33 to +29 bp to TSS. Our detailed analysis in GOLEM showed a peak of TC‐element centered −20 bp from ATG, rather than −30 bp upstream TSS as was reported in Bernard et al. (2010). Moreover, the DRE/CRT (CCGAC) element (Champ et al., 2007; Yamaguchi‐Shinozaki & Shinozaki, 1994), a CRE detected in promoter regions of several target stress‐responsive genes (Agarwal et al., 2017; Boyce et al., 2003; Knight et al., 2009; Liu et al., 1998; Vazquez‐Hernandez et al., 2017; Yang et al., 2020), exhibits a bell‐shaped peak downstream of the TSS but a sharp peak downstream of the ATG using GOLEM. This pattern suggests that the DRE/CRT element is preferentially located in the gene bodies, downstream of ATG, rather than in promoters of genes that exhibit higher expression under non‐stressed conditions in *A. thaliana*. Similarly, the ABRE motif (ACGTG), which is involved in the abscisic acid (ABA) responsiveness (Hattori et al., 2002), shows a sharp peak upstream of the TSS but a more bell‐shaped peak upstream of the ATG in *A. thaliana*, as was previously suggested in *O. sativa* aleurone cells (Watanabe et al., 2017).

### GOLEM reveals that TATA‐box‐containing promoters are associated with late pollen development

Gene expression is primarily controlled through the specific binding of various proteins to diverse DNA sequence motifs upstream/downstream of the TSS (Shiu et al., 2005). TATA‐box is a particularly well‐conserved, preferentially located motif since it is found in the same promoter region in both plants and animals. In *A. thaliana* and *O. sativa* genomes, the TATA‐box is strictly located within the −39, −26 region upstream of the TSS (Bernard et al., 2010). Even though the TATA‐box is a common CRE, it is not a general feature of all promoters (Basehoar et al., 2004; Bernard et al., 2010; Civán & Svec, 2009; Savinkova et al., 2023; Yang et al., 2007). Moreover, the percentage of the genes with TATA‐box is associated with the level of the expression. In barley embryos, the TATA‐box‐containing promoters are associated mostly with genes exhibiting high expression levels, while promoters lacking a distinct TATA‐box tend to exhibit lower expression. The genes regulated by the TATA‐box promoters were annotated as responsive to environmental stimuli, stress, and signals related to hormonal, developmental, and organ growth process levels (Pavlu et al., 2024).

**Figure 2:**
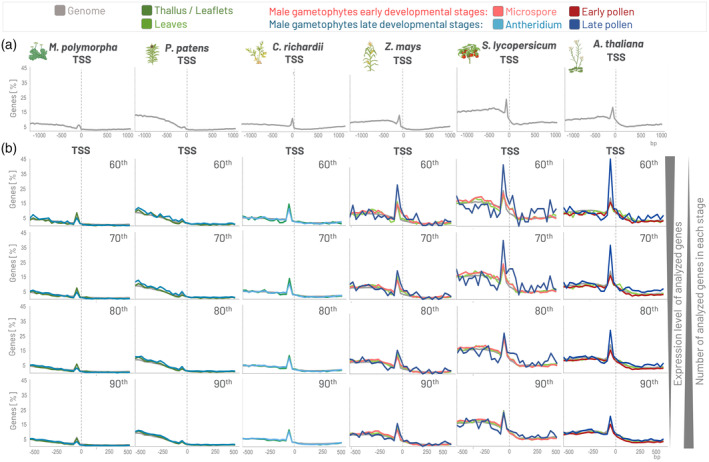
Example of the distribution of TATA‐box in genomes across plant evolution and in genes expressed at various levels in male gametophyte tissues. (a) Distribution of the TATA‐box shows that only a small fraction of plant genes actually harbor a TATA‐box in their promoters in −30 to −25 area upstream of TSS, regardless of the transcription of those genes (genome). The motif was searched in the interval <−1000, 1000> bp from TSS within the bucket size 30 bp, and the axis size was adjusted to 45% in all species. (b) Genes whose transcripts represent 60, 70, 80 and 90% of all transcripts transcribed from the total number of protein‐coding genes (60^th^, 70^th^, 80^th^ and 90^th^ percentiles) were analyzed in various selected stages in *Marchantia polymorpha*, *Physcomitrium patens*, *Ceratopteris richardii*, *Zea mays*, *Solanum lycopersicum* and *Arabidopsis thaliana*. Genes highly transcribed during late male gametophyte development in flowering plants possess a higher percentage of the TATA‐box motifs located upstream of TSS than genes transcribed during early pollen. The motif was searched in the interval <−500, 500> bp from TSS within the bucket size 30 bp, and the axis size was adjusted to 45% in all species.

Our GOLEM program allowed us easily to verify that only a small fraction of plant genes actually harbor a TATA‐box (TATAWA) in their promoter in −30 to −25 area upstream of TSS, regardless of the transcription of those genes (Figure 2a), as seen in the plant species with annotated TSS positions. Even though in bryophytes, such as the liverwort (*M. polymorpha*) and mosses (*P. patens*) the percentage of the genes with the TATA‐box upstream of TSS is nearly negligible, those genes also show preferential location in −30 to −25 area upstream of TSS.

Further, we analyzed the genes whose transcripts represent 60, 70, 80 and 90% of all transcripts transcribed from the total number of protein‐coding genes (60^th^, 70^th^, 80^th^ and 90^th^ percentiles) in various stages during the male gametophyte development and in the thallus/leaflets/leaves. Analysis of the promoters showed that TATA‐box‐containing promoters are associated with the genes expressed during the late pollen development, but not early pollen development, in flowering plants, especially in *S. lycopersicum* and *A. thaliana* (Figure 2b).

### GOLEM demonstrates distribution patterns of gene regulatory motifs linked to the male gametophyte

Unlike the position independence seen for many animal DNA sequence motifs, the activity of flowering plant DNA sequence motifs is strongly dependent on their position relative to the TSS (Voichek et al., 2024). Achieving precise localization and visualization of CRE regulatory motifs in genes that exhibit high transcription levels in various stages of male gametophyte development may help to elucidate the gene regulation in specific tissues.

**Figure 3:**
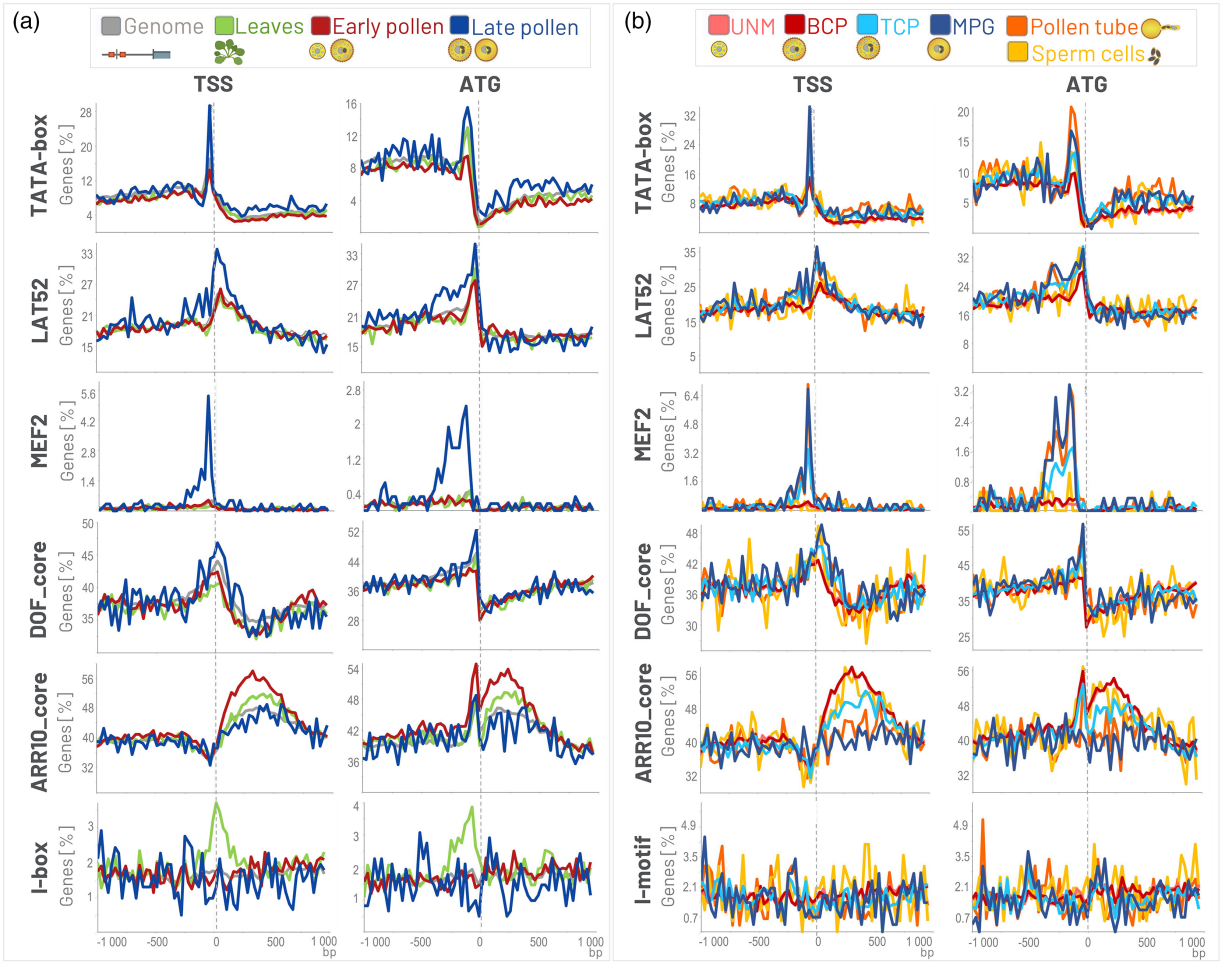
Example of the distribution of various motifs in the vicinity of TSS and ATG in *Arabidopsis thaliana*. Colored lines represent different datasets and indicate the percentage of genes with the motifs at specific positions in the promoters of the genes whose transcripts represent 80% of all transcripts transcribed from the total number of protein‐coding genes in each selected stage: (a) in early pollen, late pollen, leaves, and regardless of the transcription level (genome); (b) in UNM, BCP, TCP, MPG, pollen tube, and sperm cells. The motifs were searched in the interval <−1000, 1000> bp, within the bucket size 30 bp, and the axis size was adjusted. TATA‐box (TATAWA); LAT52 (POLLEN1_LeLAT52, AGAAA); MEF2 (CTAWWWWTAG); DOF_core (AAAG); ARR10_core (GATY); I‐motif (GATAAG); ATG, translation start site; bp, base pair; BCP, bicellular pollen; early pollen, UNM + BCP; late pollen, TCP + MPG; MPG, mature pollen grain; Pollen tube, semi‐*in vivo* grown pollen tube; TCP, tricellular pollen; TSS, transcription start site; UNM, uninucleate microspore.

LAT52 (also named POLLEN1_LeLAT52) is a pollen‐specific motif (AGAAA) recognized within the promoter of *S. lycopersicum lat52* gene that encodes an essential protein expressed in the vegetative cell during pollen maturation. It specifically directs the transcription of genes required for PT growth and fertilization, ensuring successful reproduction in flowering plants (Bate & Twell, 1998; Muschietti et al., 1994). Our analysis revealed that the preferential position of the LAT52 motif (the top of the peak) is located downstream of the TSS and upstream to ATG, i.e., in 5′UTR. Moreover, the number of genes containing the LAT52 in *A. thaliana* is higher within the 5′UTR region of genes exhibiting elevated expression (80^th^ percentile) in late pollen development and PT, as opposed to genes expressed during early pollen development or in leaves, as was expected (Figure 3).

MEF2‐type CArG‐box (CTA(A/T)_4_TAG) is bound by MADS‐protein complexes functioning in mature pollen (Shore & Sharrocks, 1995; Verelst et al., 2007). It was shown that MEF2‐type boxes are strongly overrepresented in the proximal region of promoters that are activated during the last stages of pollen development (Verelst et al., 2007). Our analysis using the GOLEM program verified a strong overrepresentation of MEF2‐type box in late pollen development in *A. thaliana* (Figure 3a), especially in genes expressed in MPG and pollen tube, but not in sperm cells or UNM/BCP (Figure 3b). The MEF2‐type box present in the promoters of the genes expressed during the late pollen development is located −80 bp upstream of the TSS.

The DOF motif is recognized by plant‐specific DNA‐binding TFs named Dof (DNA‐binding with One Finger) domain proteins (Li et al., 2014; Yanagisawa, 2002), which have crucial roles in many physiological processes, including hormone signaling and various biotic or abiotic stress responses, but are also reported to regulate many biological processes, such as dormancy or tissue differentiation (reviewed in Zou & Sun, 2023). Using the program GOLEM, we have revealed the overrepresentation of the DOF_core motif (AAAG) in genes activated during the last stages of pollen development (TCP, MPG) and in the sperm cell, compared to early pollen stages or leaves. Interestingly, the peak of the DOF_core motif is centered −40 bp upstream of ATG in *A. thaliana*, predominantly located in the 5′UTR (Figure 3a,b).

The ARR10_core motif is recognized by the ARR10 protein. ARR10 is one of a type‐B Arabidopsis Response Regulators (ARRs) TFs that are associated with the cytokinin transcriptional response network (Hosoda et al., 2002; Xie et al., 2018). Cytokinins play a crucial role in regulating reproductive development in *Arabidopsis* (reviewed in Terceros et al., 2020). Our analysis showed that the ARR10_core (GATY) motif is overrepresented in early pollen stages (UNM, BCP) and sperm cells; however, its presence is decreased in TCP and even more so in MPG stages in *A. thaliana*. The ARR10_core motifs are present not only in 5′UTRs but also within the gene bodies (Figure 3).

### GOLEM disclose localization of gene regulatory motifs in sporophyte

Although our software, GOLEM, is primarily focused on tissues associated with male gametophyte development, it can also be utilized to search for gene regulatory motifs near the TSS and ATG in leaves, leaflets, and thallus of plant species available in GOLEM. Thus, it can visualize motifs associated with the regulation of genes involved not only in gametophyte development but also in sporophyte development in angiosperms.

The I‐box has been suggested to be involved in light‐regulated and/or leaf‐specific gene expression of photosynthetic genes (Castresana et al., 1987; Gidoni et al., 1989; Manzara et al., 1991) and can be bound by myb‐like proteins in *S. lycopersicum* (Rose et al., 1999). The leaf‐specific overrepresentation of I‐box (GATAAG; Table S3) can also be tracked using the GOLEM program. The I‐box is overrepresented in the 5′UTR region of genes expressed in the sporophyte but not in the gametophyte, as detected not only in the exemplified *A. thaliana* (Figure 3) but also in *S. lycopersicum* and *Z. mays* using the GOLEM program (data not shown‐see in GOLEM program).

The DRE/CRT element is recognized by the drought‐responsive element binding (DREB) and Ethylene Response Factors (ERF), both belonging to the APETALA2/Ethylene Response Factor (AP2/ERF) family of TFs (Champ et al., 2007; Yamaguchi‐Shinozaki & Shinozaki, 1994). These cis‐elements are located in promoter regions of target stress‐responsive genes and play an important role in the regulation of stress‐inducible transcription (Agarwal et al., 2017; Yang et al., 2020). Therefore, it is not surprising that there are no significant changes in the overrepresentation of this element between sporophyte and gametophyte tissues that were not stressed, as expected. Interestingly, contrary to expectations, the DRE/CRT element (CCGAC) is preferentially located in the gene bodies of genes that exhibit higher expression under non‐stressed conditions, rather than in their promoters (Figure S3).

No significant changes in the overrepresentation between the gametophyte and sporophyte in *A. thaliana* were observed in other motifs present in the software. For example, the BR‐response element (CGTGYG) recognized by Brassinazole‐resistant (BZR) family plant‐specific TFs shows a negligible difference between sporophyte and gametophyte (Figure S3), even though BZRs play a significant role in regulating plant growth and development, as well as stress responses (Chen et al., 2021; Nolan et al., 2020; Wang et al., 2002). Similarly, the E‐box (enhancer box, CANNTG), which is recognized by the helix–loop–helix (bHLH) family of TFs and is important for plant growth, development, light signal transduction, and stress responses (reviewed in Hao et al., 2021), shows no difference between sporophyte and gametophyte.

### GOLEM enables evaluation of motif abundance across plant evolution

**Figure 4:**
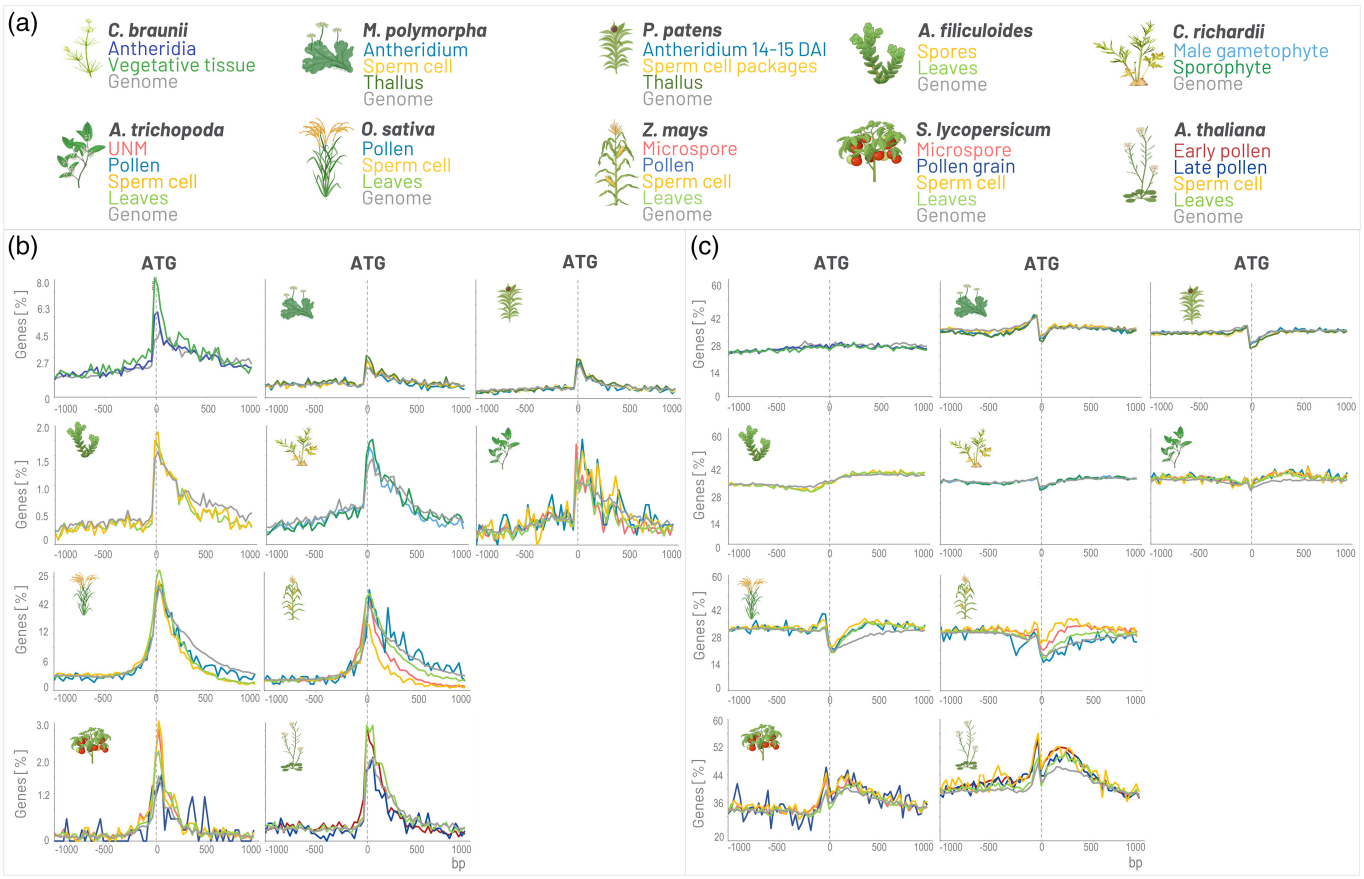
Example of the distribution of hormone‐responsive elements. (a) The genomes analyzed across plant evolution include one streptophyte alga (*Chara*), two mosses (*Marchantia* and *Physcomitrium*), two ferns (*Azolla* and *Ceratopteris*), two monocots (*Oryza* and *Zea*), and two dicots (*Solanum* and *Arabidopsis*), as well as selected tissues and developmental stages. (b) The distribution of ethylene‐responsive element (GCC‐box, GCCGCC) shows a conserved pattern downstream of ATG across the evolution, even in streptophyte algae. (c) The distribution of the ARR10_binding motif (ARR10_core, GATY) does not exhibit conserved distribution across evolution, beginning in streptophyte algae. The genes expressed in the 90^th^ percentile are shown within the range <−1000, 1000> bp, with a bucket size of 30 bp; bp, base pair. The axis size was adjusted in each row.

The GOLEM involves various plant species representing the evolution of land plants, a streptophyte algae *C. braunii* as a multicellular outgroup of land plants and model species for liverwort (*M. polymorpha*) and mosses (*P. patens*) as the early diverging groups of land plants. Further, two fern models, *C. richardii* and *A. filiculoides*, were added among those to represent vascular plants outside of seed plants. In the context of sperm cells and spores, it is worth mentioning that the two selected representatives of ferns belong to different groups based on spore morphology—*C. richardii* although aquatic, is homosporous/isosporous, whereas heterosporous *A. filiculoides* produces differentiated microspores and megaspores (Sebastian et al., 2021; Atallah & Banks, 2015). Involvement of such species enables evaluation of evolutionary conservation of tested motifs and their position in gene promoters and gene bodies (Figure 4a).

This may serve as an interesting complementary insight into the evolution of various important signaling pathways. For instance, ethylene and cytokinin signaling pathways exhibit several similarities in pathway architecture, like his‐asp phosphotransfer (Wang, Li, et al., 2020). A detailed description of both pathways and cross‐talk is out of the scope of this article; however, DNA‐binding components could be highlighted. Among others, several members of the above‐mentioned (AP2/ERF) family transcription factors, namely ERFs, are induced by ethylene and canonically recognize the GCC‐box (GCCGCC) or possibly even the DRE/CRT (CCGAC) element (Cheng et al., 2013; Sun et al., 2022; Wu et al., 2022). In the cytokinin response pathway, type‐B ARRs bind to the core sequence GATY (ARR10_core). Ethylene and cytokinin signaling pathways vividly cross‐talk on several levels (Yamoune et al., 2024; Zdarska et al., 2019), however, their evolutionary conservation differs. Components of both signaling cascades are present in land plants (Powell & Heyl, 2023), but the *C. braunii* possesses all members of the cannonical ethylene signaling pathway but not cytokinin — it is missing type‐B ARRs (Nishiyama et al., 2018).

The GCC‐box, a crucial component for ethylene‐based induction by ERF, exhibits certain conserved features across the analyzed plant representatives, indicating certain conservation within the selected taxa (Figure 4b). Such conservation is fitting the assumption that ERFs (and in extension the ethylene pathway) are present in *C. braunii* (PlantTFDB, 2022). The occurrence pattern of GCC‐box is similar in all shown species, gravitating around the start codon with higher occurrence in gene body than in 5′ UTR region. However, what is interesting, it seems that GCC‐box is over‐represented in genes of monocots. A similar trend is also observed for the DRE/CRT element (Figure S4), possibly because the transcription factors that can possibly bind DRE/CRT elements (DREBs canonically) and GCC‐boxes (canonically ERFs) belong to the same AP2/ERF family. This difference in representation could imply motif expansion and potential functional diversification of the GCC‐box and DRE/CRT element in monocots.

The ARR10_core motif, associated with components of the cytokinin response, shows higher variability in motif presence for tested taxa. Fitting the assumption that type‐B ARRs are missing in *C. braunii*, there is no distinguishable distribution enrichment pattern in gene bodies nor pomoters. However, in land plants, an enrichment prior to ATG could be observed, which is less pronounced in bryophytes like *M. polymorpha* and *P. patens* but more prevalent in presented seed plants. Looking at representatives of ferns and basal angiosperms, a similar pattern like in mosses could be observed in *C. richardii* and *A. trichopoda* but not *A. filliculoides*. In both monocot and dicot representatives, the ARR10_core motif is also enriched in gene bodies of expressed genes, as partially discussed above for *A. thaliana*. In general, it seems that there is a pattern fitting the diversification of land plants from absence in charophytes through slight enrichment in bryophytes to a specific bimodal pattern in monocots and dicots. Hypothetically, this diversification of the ARR10_core motif abundance pattern in flowering plants could correspond to the seed plant innovation in ADP/ATP‐dependent cytokinin biosynthesis and its diversification in monocots and dicots (Powell & Heyl, 2023; Wang, Lin, et al., 2020).

### Further analysis of the genes with a motif of interest in their promoters

The GOLEM program enables exporting normalized expression values of genes, depicted as TPM, from selected tissues at a specified percentile or for a chosen number of genes. This export also includes their expression levels in other tissues, formatted as a table in XLSX. Additionally, the table contains the gene identifier numbers of the genes that contain the motif of interest in a certain bucket. These gene accession numbers can be analyzed using various bioinformatics approaches, such as gene description search, gene ontology (GO) enrichment analysis, protein–protein interaction network analysis, or other relevant analyses.

**Figure 5:**
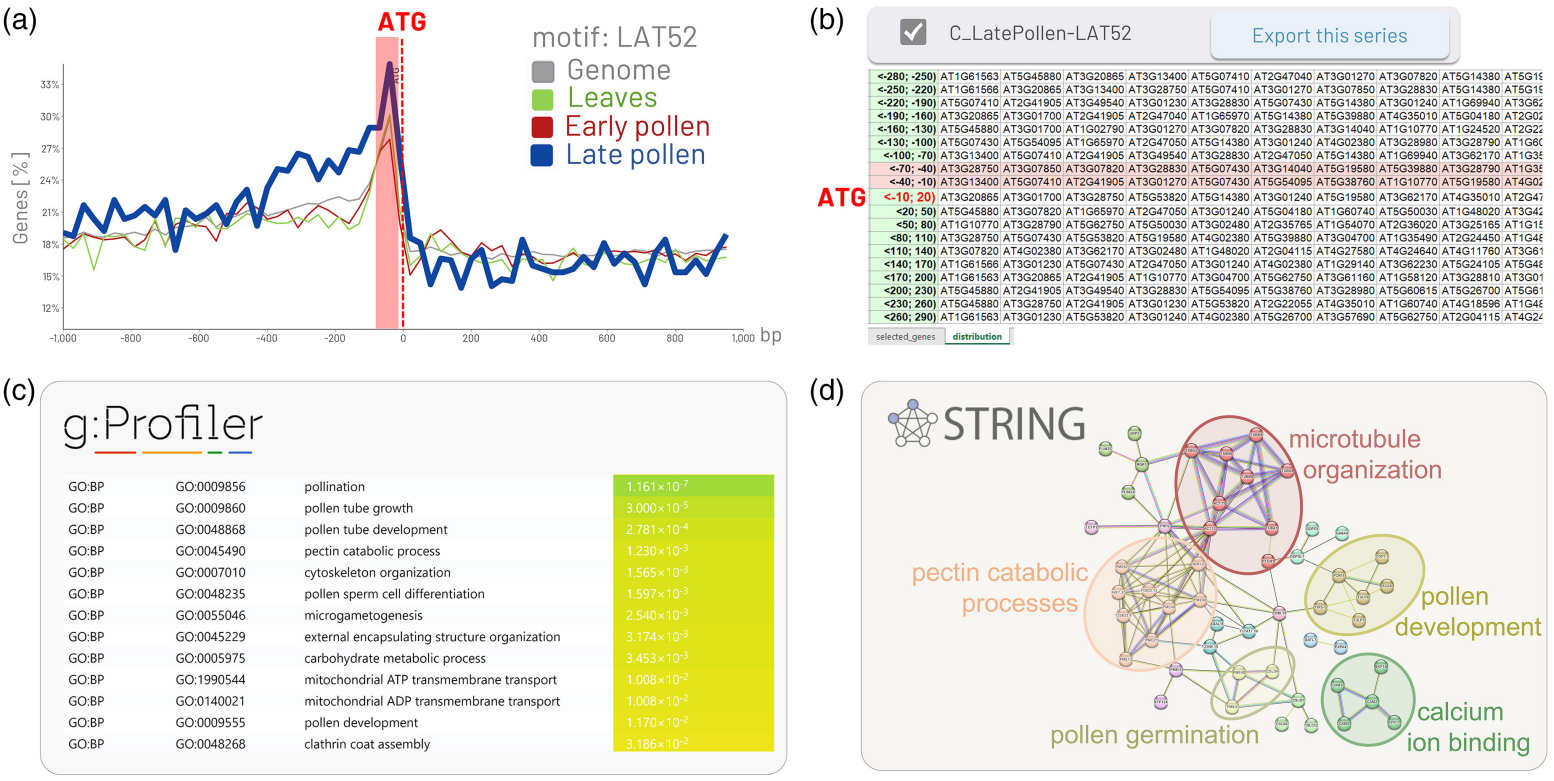
Functional analysis of the genes with LAT52 in the vicinity of ATG. (a) The genes expressed in the 80^th^ percentile during the late pollen stage in *Arabidopsis thaliana*, <−1000, 1000> bp within the bucket size 30 bp were visualized using GOLEM software. (b) The XLSX table was exported from the late pollen stage using GOLEM. (c) The gene identifier numbers from the XLSX table, covering buckets between <−70, −10> bp upstream from the ATG, were uploaded to the g:PROFILER for GO enrichment analysis. (d) The genes associated with the GO term biological processes (GO:BP) were uploaded to STRING to visualize the comprehensive network of protein–protein interactions of proteins whose genes are expressed during late pollen development and contain LAT52 upstream of ATG.

To illustrate this feature, genes containing the LAT52 motif in their promoter were exported. LAT52 shows a sharp peak upstream of the ATG in genes expressed in the 80^th^ percentile during the late pollen stage in *A. thaliana* (Figures 3 and 5a). The XLSX table was exported from the late pollen stage using GOLEM, covering buckets between −70 and −10 bp from the ATG start codon (Figure 5b). The gene identifier numbers (AGI‐Arabidopsis Genome Initiative ID numbers) from this table, within the −70 to −10 bp region of the ATG start codon, were uploaded to the g:PROFILER for GO enrichment analysis. The GO analysis revealed that genes containing the LAT52 motif in the region −70 to −10 bp of their ATG are enriched in GO terms associated with biological processes (BP) such as pollination, PT growth and development, cytoskeleton organization, pectin catabolic processes, and mitochondrial ATP/ADP transport (Figure 5c). All these terms are relevant to PT growth; for example, pectin plays a role in adhesion between the style and PT to prevent PT wandering, and cytoskeleton components like microtubules and actin filaments are involved in mitochondrial distribution in PT tip growth, as reviewed by Hafidh and Honys (2021).

When genes associated with biological processes (BP) in g:PROFILER were uploaded to STRING, a comprehensive network of predicted and known protein interactions was generated. These interactions, which include both physical and functional associations, revealed that genes containing the LAT52 motif within the region −70 to −10 bp upstream of the ATG, expressed during late pollen development in *A. thaliana*, are associated with biological processes such as pollen germination, pollen development, microtubule organization, pectin catabolic processes, and calcium ion binding (Figure 5d). All these processes are crucial for PT growth, development, and male–female communication, as reviewed by Hafidh and Honys (2021).

## CONCLUSION

Achieving accurate localization and visualization of gene regulatory motifs in promoters of genes with high transcription levels restricted to specific tissues involves a multi‐step process. This process can be hindered by the user's proficiency with various bioinformatics tools or the requirement for input data in specific formats. To address the challenge of precisely localizing and visualizing regulatory motifs near transcription and translation start sites‐key elements in gene regulation in specific tissues‐we introduced the GOLEM software. GOLEM provides a user‐friendly platform for investigating the distribution of any motif of interest in gene promoters across diverse plant genomes and developmental tissues, with a particular emphasis on male gametophytes.

Using gene regulatory motifs such as LAT52, MEF2, DOF_core, and ARR10_core‐previously implicated in the regulation of pollen and/or plant development‐we demonstrated that the GOLEM program is an effective tool for visualizing the distribution of these motifs within gene promoters. Our analysis with GOLEM revealed whether these motifs are preferentially associated with genes expressed during the early or late stages of male gametophyte development. Additionally, GOLEM enables accurately mapping the positional distribution of peaks near the TSS or ATG, even within the 5′ UTR, across various species. Beyond gametophyte‐specific motifs, GOLEM also facilitates the visualization of motifs in plant sporophytes (e.g., leaves). For instance, GOLEM has shown that I‐box motifs, which are associated with plant photosynthesis, are overrepresented in the 5′ UTR region of genes expressed in the sporophyte but not in the gametophyte. Additionally, GOLEM has revealed that the distribution of the ethylene‐responsive element, GCC‐box, exhibits a conserved pattern downstream of ATG across plant evolution, even in streptophyte algae (*C. braunii*). In contrast, the distribution of ARR10_core, associated with the cytokinin pathway, does not show such a conserved pattern across the evolution. These observations are in congruence with the fact that DNA‐binding components of both signaling cascades are present in land plants (Powell & Heyl, 2023), but *C. braunii* possesses all members of the canonical ethylene but not the cytokinin signaling pathway (Nishiyama et al., 2018). Furthermore, GOLEM allows users to track all analyses and export data on genes with motifs of interest at specific positions relative to the TSS/ATG for further analysis using tools such as Gene Ontology (GO) or STRING.

Overall, the user‐friendly online software GOLEM is a valuable resource for elucidating the abundance, distribution, and tissue‐specific association of any motif of interest across diverse plant species and evolutionary stages. As GOLEM does not require programming skills or advanced bioinformatics expertise, it is particularly well‐suited for biologists with limited experience in complex bioinformatics tools.

## MATERIALS AND METHODS

The GOLEM program is divided into two main phases: data processing pipeline and data visualization.

### Data processing pipeline

#### Segmentation of genomic sequences upstream/downstream of TSS and ATG

The reference genomes and genome annotation files from *C. braunii*, *M. polymorpha*, *P. patens*, *A. filiculoides*, *C. richardii*, *A. trichopoda*, *O. sativa*, *Z. mays*, *S. lycopersicum*, and *A. thaliana* were downloaded in the FASTA format and General Feature Format (GFF3), respectively (Table S1a). The data processing pipeline first parses location data of individual genes on a reference genome, using annotation data from a GFF3 file, to identify the position of TSS (transcription start site) and ATG (first translated codon) in the reference genome. The locations of TSS and ATG were determined as positions of “five_prime_UTR” and “start codon” in GFF3, respectively, for *Marchantia*, *Physcomitrium*, *Amborella*, *Oryza*, *Zea*, *Solanum* and *Arabidopsis*. For *Chara* and *Azolla* the “CDS” (min/max) for the genes oriented at ± DNA strand was used to determine the ATG position. For *Ceratopteris* “CDS.1” was used to identify the ATG position and “five_prime_UTR” to identify TSS. The analyzed dataset comprises a defined segment of genomic sequences specified by the user (e.g., <−1000, 1000> bp) upstream and downstream of the TSS or ATG.

#### TPM values from various plant tissues and developmental stages

The pipeline matches individual genes against a Transcript Per Million (TPM) table from various tissues and developmental stages. The TPM values express normalized transcription rates of individual genes obtained from RNA‐seq datasets. The TPM for *A. thaliana* leaves, seedling, egg, sperm, semi‐*in vivo* grown pollen tube (SIV_PT; hereafter referred to as pollen tube [PT]), and tapetum samples together with all *C. braunii*, *C. richardii* and *Z. mays* samples were processed in the following manner: the RNA‐seq datasets were downloaded as fastq files from Sequence Read Archive (SRA, https://www.ncbi.nlm.nih.gov/sra/), accession codes are stated in Table S1(b). The raw reads were checked for read quality control (Phred score cutoff 20) and trimmed of adapters with Trim Galore! (https://www.bioinformatics.babraham.ac.uk/projects/trim_galore/, v 0.5.0). Next, the reads were mapped with the use of Spliced Transcripts Alignment to a Reference (STAR) v 2.7.10a (Dobin et al., 2013) aligner to both genome and transcriptome. The TPM values were calculated from RNA‐Seq using Expectation Maximization (RSEM) (Li & Dewey, 2011) on the gene level (*Arabidopsis*, *Zea*). The TPM values of sample replicas were then averaged and used as input data for GOLEM. TPM values for *A. thaliana* early pollen stage were calculated as a mean of Uninucleate microspore (UNM) and Bicellular pollen (BCP) stages; for the late pollen stage as a mean of Tricellular pollen (TCP) and Mature pollen grain (MPG) stages. *Arabidopsis* genes were annotated by The Arabidopsis Information Resource (TAIR, https://www.arabidopsis.org/tools/bulk/genes/index.jsponarabidopsis.org, 10.10.2022), while MaizeMine v.1.5 (https://maizemine.rnet.missouri.edu/maizemine/begin.do) was used for gene annotation of *Zea*. The GFF3 files for *M. polymorpha*, *P. patens*, *A. trichopoda*, *O. sativa*, *Z. mays*, *S. lycopersicum*, and *A. thaliana* organisms were processed with the AGAT analysis toolkit (https://zenodo.org/record/7255559#.ZAn_kS2ZPfY, v1.0.0) prior to their usage in GOLEM. The TPM values for *M. polymorpha*, *P. patens*, *A. filiculoides*, *A. trichopoda*, *O. sativa*, and *S. lycopersicum* tissue were acquired from the Conekt database (https://conekt.sbs.ntu.edu.sg, Julca et al., 2021). The TPM values for pollen developmental stages of *A. thaliana* Columbia‐0 (Col‐0) and Landsberg erecta (Ler) were extracted from Klodová et al. (2023). The normalized TPM values used for the data processing pipeline are listed in Table S2.

#### Individual gene matching against TPMs

The pipeline matches individual genes against a TPM table from various tissues and developmental stages (hereafter referred to as stages). The sequential values of a series of TPM numbers are pooled. The output of this data processing pipeline is a separate FASTA‐compatible file that contains each valid gene from the original input, along with information about the position of TSS and ATG, and transcription rates (TPMs) in each stage added as comments. The pipeline also generates a validation log that provides information about genes that were excluded, that is, non‐protein coding genes (noStartCodonFound), pseudogenes (noFivePrimeUtrFound, noTpmDataFound), and genes without TSS (noFivePrimeUtrFound, if relevant for certain organisms). In *C. braunii*, *A. filiculoides*, *A. trichopoda* and *O. sativa*, the GFF3 gene annotation of TSS is inadequate, limiting the search to motifs in the vicinity of the ATG.

#### Motif search

Motif search uses regular expressions to search the input string of base pairs. For each motif, the reverse complement is calculated and then translated together with the forward strand into regular expressions. When the regular expression is run against the input data, we record all results and calculate their relative positions (adjusted to the middle of the motif) relative to TSS and ATG. The motif sequences are searched in the buckets that can be specified by the user (default size is 30 bp).

### Data visualization

The data visualization phase consists of five steps, as depicted in Figure 1 and Figure S1. In the selected genome (Figure 1a), the user can choose the genomic interval to be searched, effectively specifying the window of the sequence where the search is performed, and focus on a defined region in the vicinity of the TSS or ATG (Figure 1b; Figure S1b). A single custom motif or multiple motifs, including degenerate motifs of interest, can be defined by users (Figure 1c; Figure S1c). Optionally, the motif can be chosen from several motifs present in the software: (i) conserved eukaryotic promoter motif: TATA‐box (TATAWA; Feng et al., 2016; Lifton et al., 1978; Suzuki et al., 2001); (ii) motifs associated with pollen development: pollen Q‐element (AGGTCA; Hamilton et al., 1998); POLLEN1_LeLAT52 (AGAAA; Bate & Twell, 1998; Muschietti et al., 1994); CAAT‐box (CCAATT; Peng et al., 2017); GTGA motif (GTGA; Rogers et al., 2001); (iii) motifs associated with plant hormone‐mediated regulation: ABRE motif (ACGTG; Hattori et al., 2002; Watanabe et al., 2017); ARR10_core (GATY; Hosoda et al., 2002; Xie et al., 2018); E‐box (CANNTG; reviewed in Hao et al., 2021); G‐box (CACGTG; Shen & Ho, 1995; Yamaguchi‐Shinozaki et al., 1990); GCC‐box (GCCGCC; Ohme‐Takagi & Shinshi, 1995; Zhang et al., 2004); (iv) biotic and abiotic stress responses: BR_response element (CGTGYG; Chen et al., 2021; Nolan et al., 2020; Wang et al., 2002); DOF core motif (AAAG; Li et al., 2014; Yanagisawa, 2002; reviewed in Zou & Sun, 2023); DRE/CRT element (CCGAC; Agarwal et al., 2017; Yamaguchi‐Shinozaki & Shinozaki, 1994; Yang et al., 2020); and (v) motifs regulated by light: I‐box (GATAAG; Castresana et al., 1987; Gidoni et al., 1989). A table of motifs selected to demonstrate the functionality of the GOLEM program is also provided in Table S3.

Motifs are searched in both forward and reverse forms, and the reverse form is calculated automatically (Figure S1d). Before the entire analysis, the user confirms the stages to be searched (Figure 1d; Figure S1e) and selects the method for choosing genes for analysis (Figure S1f). Gene selection, based on TPM, uses a given percentile (genes whose transcripts will represent e.g., 90% of all transcripts transcribed from the total number of protein‐coding genes in each selected stage; default is the 90^th^ percentile) to select the genes that are the most/least transcribed in tissues or developmental stages of interest to exclude the genes with low or even negligible transcription. The number of selected genes within the given percentile can be tracked during the proceeding steps. In addition to selecting genes based on a specified percentile of transcription levels, users can choose a specific number of genes with the highest or lowest transcription levels for analysis. The motif distribution, regardless of the transcription level, can also be included in the analysis (stage genome, hereafter referred to as “all”).

The goal of the analysis is to visualize the distribution of motifs of interest in the vicinity of the TSS or ATG of all protein‐coding genes, or exclusively in selected genes that exhibit high/low transcription levels in particular tissues and developmental stages (Figure 1e; Figure S1g). Results are presented graphically, with each stage color‐coded, and can be displayed as percentages of the genes with a certain motif or as simple counts. The user can also choose to display either the number of motifs found or aggregate the motifs by genes (Figure S1h).

Additionally, the set of genes with specific motifs at defined positions can be exported for further analysis (Figure 1f; Figure S1i,j). Within the analysis, the application allows the user to export each data series or export the aggregated data for all data series in XLSX format. The user can also see the distribution of individual motifs and drill down through them.

The data visualization phase involves an application written in Flutter/Dart (Meiller, 2021), which can be run as a standalone application or compiled into JavaScript and hosted on the web as a single‐page web app (https://golem.ncbr.muni.cz).

### Limitations

The entire processing takes place on the client within the application or web browser, with input files loaded into memory. The program's ability to work with large datasets may be constrained by the available memory and the web browser's local client settings. Nevertheless, we found that even in the web application, where performance is limited due to the inefficiencies of JavaScript compared to platform‐native code, performance is satisfactory on modern computers without the need for significant code optimization. These obstacles can be seen, for example, in the analysis of large genomes such as *O. sativa*.

### Gene ontology annotation and functional analysis

To interpret Gene Ontology (GO), genes containing the motif of interest (LAT52) located between −70 and −10 bp from the ATG start codon and expressed in the 80^th^ percentile during the late pollen stage in *A. thaliana* were exported from the GOLEM in an XLSX table. These genes were identified by GOLEM within the interval <−1000, 1000> bp relative to the ATG, using a 30 bp bucket size. The list of AGI locus codes (gene identifiers) was exported and then uploaded to the g:PROFILER software (Kolberg et al., 2023; Reimand et al., 2007) for functional annotation analysis, using default parameters. The functional annotation covered biological processes (BP), cellular compartments, and molecular functions. Further, the genes listed under BP in g:PROFILER were uploaded to the Search Tool for the Retrieval of Interacting Genes/Proteins (STRING, https://string‐db.org, Snel et al., 2000; Szklarczyk et al., 2023), where genes associated with the same biological processes were clustered and highlighted.

## AUTHOR CONTRIBUTIONS

PPS and DH conceived the study. BK and JR analyzed RNA‐seq data and calculated the TPM. LN imposed a computation analysis and visualization tool. TR and RS implemented the website and supported program accessibility. TP and AK helped with the analysis of exported data. PPS wrote the paper with the help of all co‐authors.

## CONFLICT OF INTEREST

The authors report no declarations of interest.

## Supporting information


**Figure S1.** A detailed overview of the workflow of the GOLEM software. (a) One plant species across the plant Tree of Life is chosen, and the data are downloaded on the web browser (*Chara*, *Marchantia*, *Physcomitrium*, *Amborella*, *Azolla*, *Ceratopteris*, *Oryza*, *Zea*, *Solanum*, and *Arabidopsis*). If available, the positions of both TSS and ATG are given. (b) The defined region (genomic interval) in the vicinity of the TSS or ATG, within the selected bucket size (bp), is chosen. (c) A single custom motif of interest can be defined by users. Additionally, multiple motifs as well as degenerate motifs can also be searched for by users. (d) Optionally, the motif can be chosen from several motifs present in the software. (e) The promoters of genes showing expression in selected tissues and developmental stages (sporophyte, male gametophyte), along with an analysis of genome‐wide distribution regardless of transcription, are chosen for the analysis. (f) The selection of genes that are highly or minimally transcribed in tissues or developmental stages of interest can be determined by the user, based on a specified percentile (default is the 90^th^ percentile) or a certain number of genes included in the analysis. (g) The exemplified “My own motif, TC‐element and ARR10_core” motifs show various distributions upstream/downstream of TSS. The motif TC‐element shows higher prevalence in the promoters of genes transcribed during late pollen development (blue), and the motif ARR10_core shows higher prevalence in the promoters of the genes transcribed during early pollen (red) stages, in comparison to the genome‐wide distribution (all; gray). The symbol (=) is used to change the curve order. The individual stages can be made invisible. (h) The customization options for the output graph include adjusting curve color/stroke, axes size, and displaying either percentages or counts of genes with the motifs of interest. Additionally, the output graph can be saved in PNG format. (i) The accession numbers of genes with certain motif at the selected interval may be exported in XLSX format tables. (j) The normalized expression values of genes, represented as Transcript Per Million (TPM) in selected tissue at a specified percentile or for a chosen number of genes, along with their expression in other tissues, can be exported as a table in XLSX format. Some plant icons were created with BioRender.com.
**Figure S2.** The number of genes contributing to expression programs varies between developmental stages or tissues. (a) In early pollen stages, leaves and seedlings of *Arabidopsis thaliana*, the genes whose transcripts account for 90% of all transcripts transcribed from the total number of protein‐coding genes (90^th^ percentile) represent 27, 24 and 30% of the total protein‐coding genes, respectively. In late pollen stages, sperm cells and PT, those genes represent 7, 3 and 5%, respectively. (b) In bryophyte *Marchantia polymorpha*, the genes whose transcripts comprise 90^th^ percentile show more similar levels in antheridia, sperm cells and thallus, 25, 30 and 29%, respectively. UNM, uninucleate microspore; BCP, bicellular pollen; early pollen, UNM + BCP; TCP, tricellular pollen; MPG, mature pollen grain; late pollen, TCP + MPG; PT, semi‐*in vivo* grown pollen tube; Percentile: genes whose transcripts represent a certain percent of all transcripts transcribed from the total number of protein‐coding genes in each selected stage.
**Figure S3.** Example of the distribution of various motifs in the vicinity of TSS and ATG in *Arabidopsis thaliana* with a focus on plant leaves and seedlings. Colored lines represent different datasets and indicate the percentage of genes containing selected motifs at specific positions in the promoters of the genes whose transcripts represent 80% of all transcripts transcribed from the total number of protein‐coding genes in each selected stage: early pollen, late pollen, leaves, and seedling regardless of the transcription level (genome). The motifs were searched in the interval <−1000, 1000> bp, within the bucket size 30 bp, and the axis size was adjusted. I‐motif (GATAAG); ABRE (ACGTG); TC_element (TCTTCT, TTTCTT, TTCTTC); DRE/CRT_element (CANNTG); BR_response element (CGTGYG); TSS, transcription start site; ATG, translation start site; early pollen, UNM + BCP; late pollen, TCP + MPG; bp, base pair.
**Figure S4.** Example of the distribution of the DRE/CRT element. (a) The genomes analyzed across plant evolution include one streptophyte alga (*Chara*), two mosses (*Marchantia* and *Physcomitrium*), two ferns (*Azolla* and *Ceratopteris*), two monocots (*Oryza* and *Zea*), and two dicots (*Solanum* and *Arabidopsis*), as well as selected tissues and developmental stages. (b) The distribution of the DRE/CRT (CCGAC) element shows a gravitating around the start codon with a higher occurrence in the gene body than in the 5′ UTR region. This element seems to be over‐represented in genes of monocots. The genes expressed in the 90^th^ percentile are shown within the range <−1000, 1000> bp, with a bucket size of 30 bp; bp, base pair. The axis size was adjusted in each row.

**Table S1.** (a) The reference genomes and genome annotation files used in GOLEM software. (b) The tissues/male gametophyte developmental stages present in GOLEM software, along with the source of Transcript Per Million (TPM) values or RNA‐seq datasets used for their calculation.

**Table S2.** Normalized TPM values used for the data processing pipeline.

**Table S3.** Motifs selected for the GOLEM program.

## Data Availability

Availability and implementation: GOLEM is freely available at https://golem.ncbr.muni.cz and its source codes are provided under the MIT license at GitHub at https://github.com/sb‐ncbr/golem.
