# Supplementary material for: GOLEM: A tool for visualizing the distribution of Gene regulatOry eLEMents within the plant promoters with a focus on male gametophyte
Source: Plant J. 2025 Mar 2;121(5):e70037. doi: 10.1111/tpj.70037 (PMC11873679; doi:10.1111/tpj.70037)
Supplement: Supplementary file 1 — Figure S1. A detailed overview of the workflow of the GOLEM software. (a) One plant species across the plant Tree of Life is chosen, and the data are downloaded on the web browser (Chara, Marchantia, Physcomitrium, Amborella, Azolla, Ceratopteris, Oryza, Zea, Solanum, and Arabidopsis). If available, the positions of both TSS and ATG are given. (b) The defined region (genomic interval) in the vicinity of the TSS or ATG, within the selected bucket size (bp), is chosen. (c) A single custom motif of interest can be defined by users. Additionally, multiple motifs as well as degenerate motifs can also be searched for by users. (d) Optionally, the motif can be chosen from several motifs present in the software. (e) The promoters of genes showing expression in selected tissues and developmental stages (sporophyte, male gametophyte), along with an analysis of genome‐wide distribution regardless of transcription, are chosen for the analysis. (f) The selection of genes that are highly or minimally transcribed in tissues or developmental stages of interest can be determined by the user, based on a specified percentile (default is the 90th percentile) or a certain number of genes included in the analysis. (g) The exemplified “My own motif, TC‐element and ARR10_core” motifs show various distributions upstream/downstream of TSS. The motif TC‐element shows higher prevalence in the promoters of genes transcribed during late pollen development (blue), and the motif ARR10_core shows higher prevalence in the promoters of the genes transcribed during early pollen (red) stages, in comparison to the genome‐wide distribution (all; gray). The symbol (=) is used to change the curve order. The individual stages can be made invisible. (h) The customization options for the output graph include adjusting curve color/stroke, axes size, and displaying either percentages or counts of genes with the motifs of interest. Additionally, the output graph can be saved in PNG format. (i) The accession numb [file TPJ-121-0-s003.pdf]

Supplementary Material

GOLEM: A tool for visualizing the distribution of Gene regulatOry eLEMents within the plant promoters with a focus on male gametophyte

Lukáš Nevosád[1], Božena Klodová[2], Jiří Rudolf[1,3], Tomáš Raček[1,3], Tereza Přerovská[1,3], Alžbeta Kusová[1,3], Radka Svobodová[1,3], David Honys[2], and Petra Procházková Schrumpfová[1,3*]

[1]National Centre for Biomolecular Research, Faculty of Science, Masaryk University, Kotlářská 2, 611 37 Brno, Czech Republic

[2]Laboratory of Pollen Biology, Institute of Experimental Botany of the Czech Academy of Sciences, Rozvojová 263, 165 02 Prague, Czech Republic

[3]Central European Institute of Technology, Masaryk University, Kamenice 5, 625 00 Brno, Czech Republic

*corresponding author

schpetra@sci.muni.cz

Figures

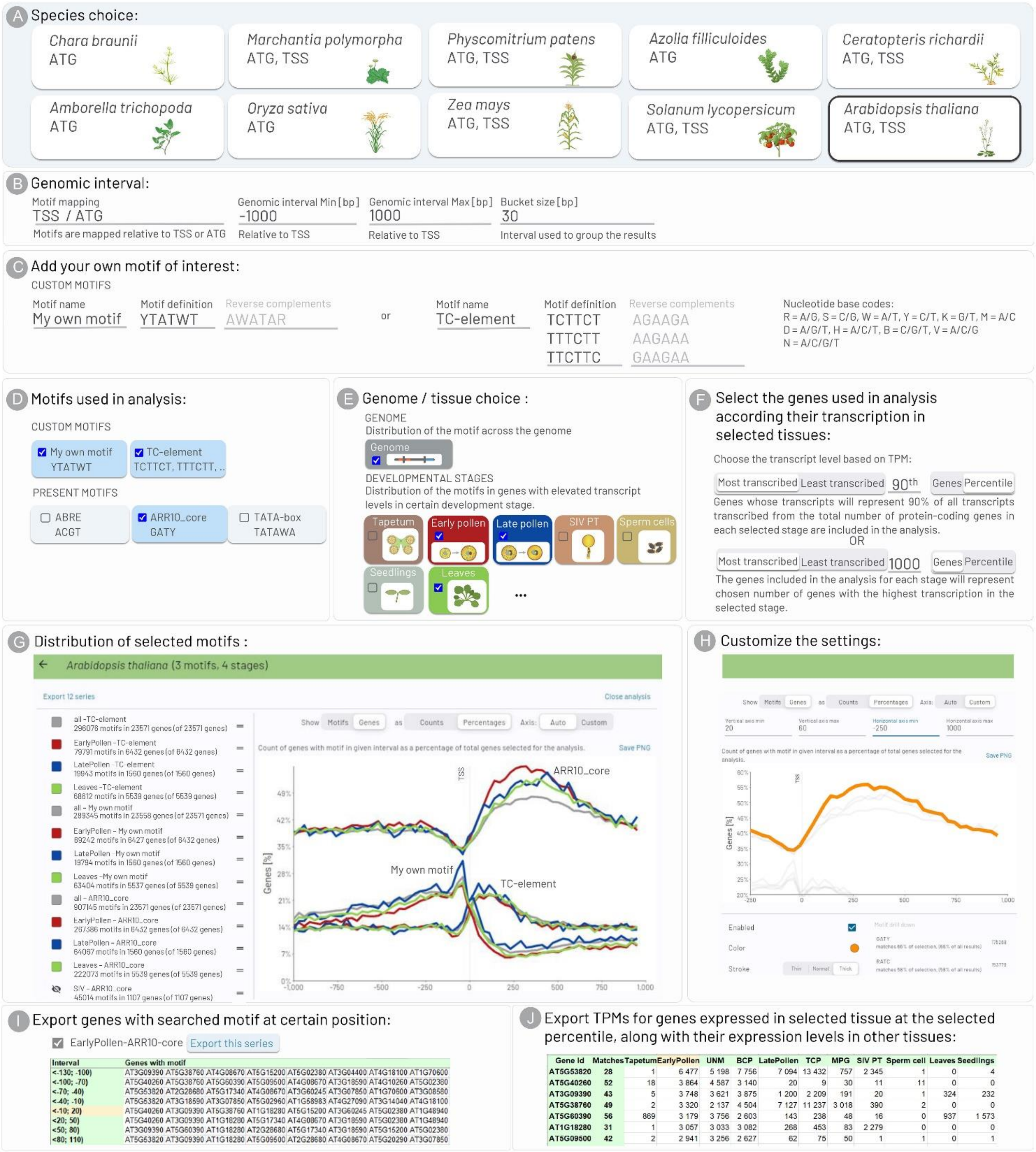

Figure S1. A detailed overview of the workflow of the GOLEM software.

(a) One plant species across the plant Tree of Life is chosen, and the data are downloaded on the web browser (*Chara, Marchantia*, *Physcomitrium*, *Amborella*, *Azolla, Ceratopteris*, *Oryza*, *Zea*, *Solanum*, and *Arabidopsis*). If available, the positions of both TSS and ATG are given.

(b) The defined region (genomic interval) in the vicinity of the TSS or ATG, within the selected bucket size (bp), is chosen.

(c) A single custom motif of interest can be defined by users. Additionally, multiple motifs as well as degenerate motifs can also be searched for by users.

(d) Optionally the motif can be chosen from several motifs present in the software.
(e) The promoters of genes showing expression in selected tissues and developmental stages (sporophyte, male gametophyte), along with an analysis of genome-wide distribution regardless of transcription, are chosen for the analysis.
(f) The selection of genes that are highly or minimally transcribed in tissues or developmental stages of interest can be determined by the user, based on a specified percentile (default is the 90th percentile) or a certain number of genes included in the analysis.
(g) The exemplified “My own motif, TC-element and ARR10_core” motifs show various distributions upstream/downstream of TSS. The motif TC-element shows higher prevalence in the promoters of genes transcribed during Late pollen development (blue) and the motif ARR10_core shows higher prevalence in the promoters of the genes transcribed during early pollen (red) stages, in comparison to the genome-wide distribution (all; grey). The symbol (=) is used to change the curve order. The individual stages can be made invisible.
(h) The customization options for the output graph include adjusting curve color/stroke, axes size, and displaying either percentages or counts of genes with the motif of interest. Additionally, the output graph can be saved in PNG format.
(i) The accession numbers of genes with certain motif at the selected interval may be exported in XLSX format tables.
(j) The normalized expression values of genes, represented as Transcript Per Million (TPM) in selected tissue at a specified percentile or for a chosen number of genes, along with their expression in other tissues, can be exported as a table in XLSX format. Some plant icons were created with BioeRender.com.

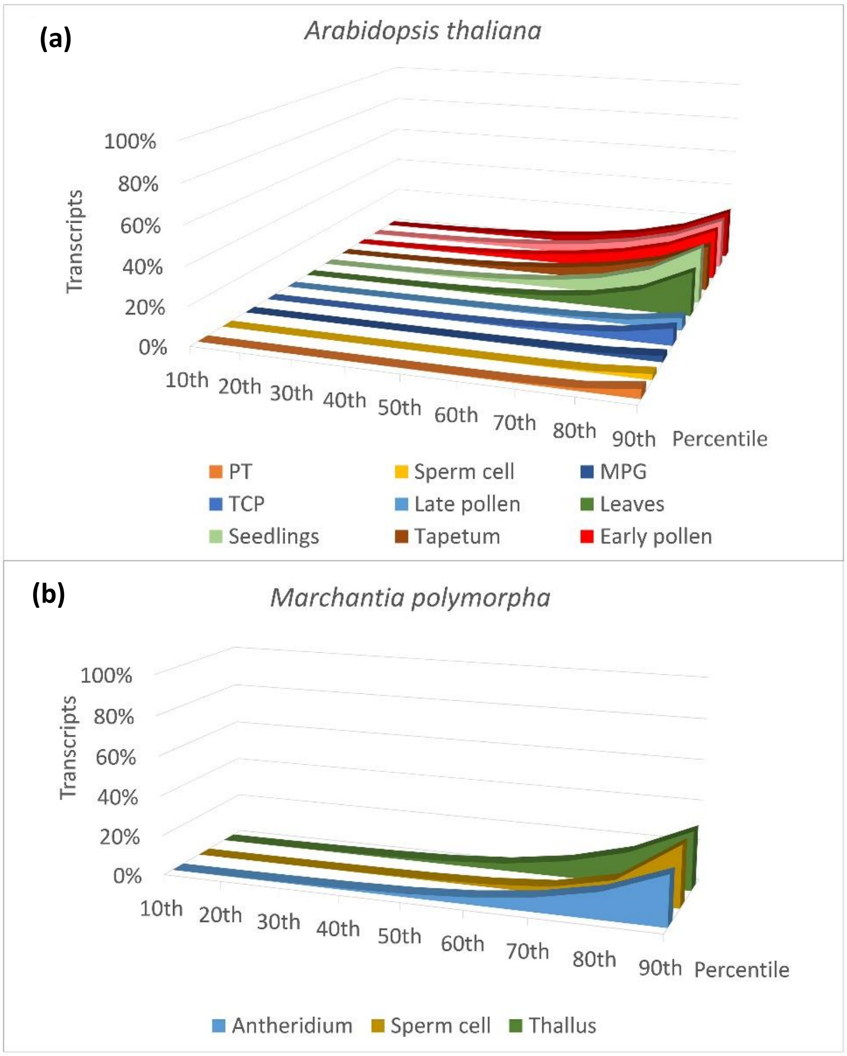

Figure S2. The number of genes contributing to expression programs varies between developmental stages or tissues.

(a) In early pollen stages, leaves and seedlings of *A. thaliana* the genes whose transcripts account for 90% of all transcripts transcribed from the total number of protein-coding genes (90th percentile) represent 27%, 24% and 30% of the total protein-coding genes, respectively. In late pollen stages, sperm cells and PT, those genes represent 7%, 3% and 5%, respectively.

(b) In bryophyte *M. polymorpha*, the genes whose transcripts comprise 90th percentile show more similar levels in antheridia, sperm cells and thallus, 25%, 30% and 29%, respectively. UNM, uninucleate microspore; BCP, bicellular pollen; early pollen, UNM + BCP; TCP, tricellular pollen; MPG, mature pollen grain; late pollen, TCP + MPG; PT, semi-in vivo grown pollen tube; Percentile, genes whose transcripts represent certain percent of all transcripts transcribed from the total number of protein-coding genes in each selected stage.

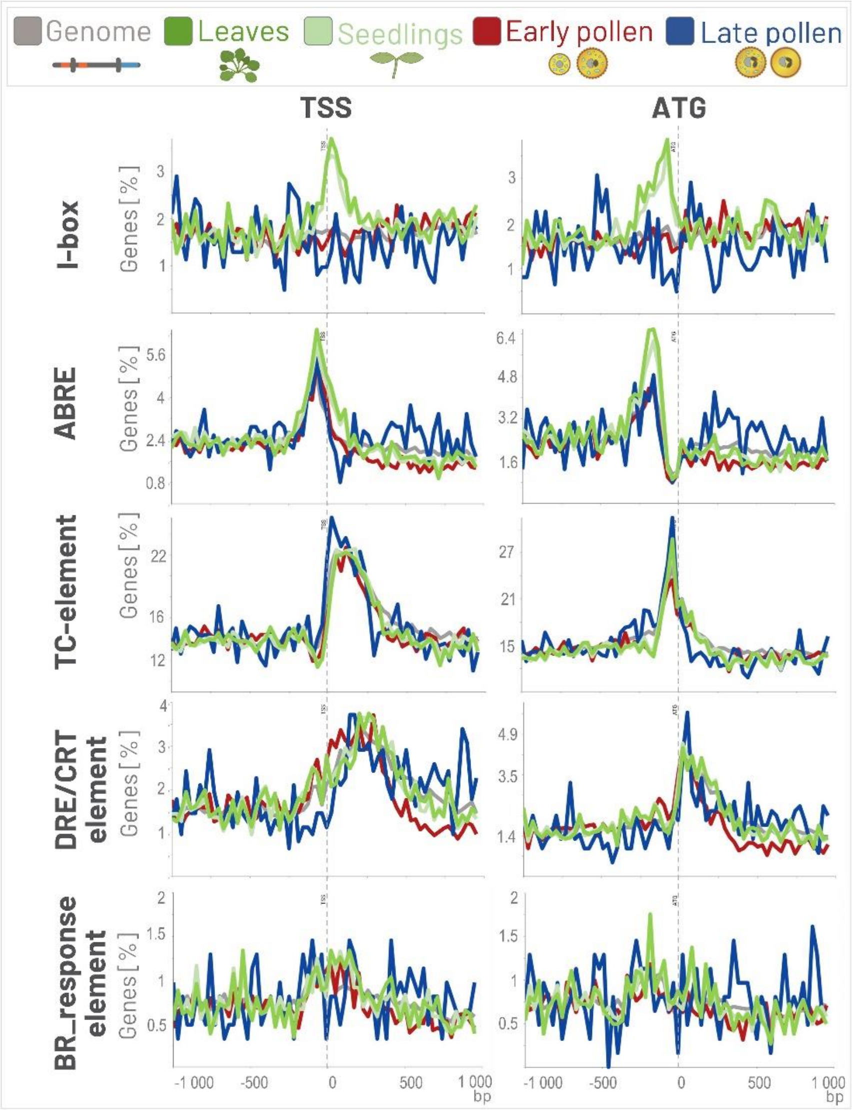

Figure S3. Example of the distribution of various motifs in the vicinity of TSS and ATG in *A. thaliana* with a focus on plant leaves and seedlings. Colored lines represent different datasets and indicate the percentage of genes containing selected motifs at specific positions in the promoters of the genes whose transcripts represent 80% of all transcripts transcribed from the total number of protein-coding genes in each selected stage: early pollen, late pollen, leaves, seedling and regardless of the transcription level (genome). The motifs were searched in the interval <-1000, 1000> bp, within the bucket size 30 bp, and the axis size was adjusted. I-motif (GATAAG); ABRE (ACGTG); TC_element (TCTTCT, TTTCTT, TTCTTC); DRE/CRT_element (CANNTG); BR_response element (CGTGYG); TSS, transcription start site; ATG, translation start site; early pollen, UNM + BCP; late pollen, TCP + MPG; bp, base pair.

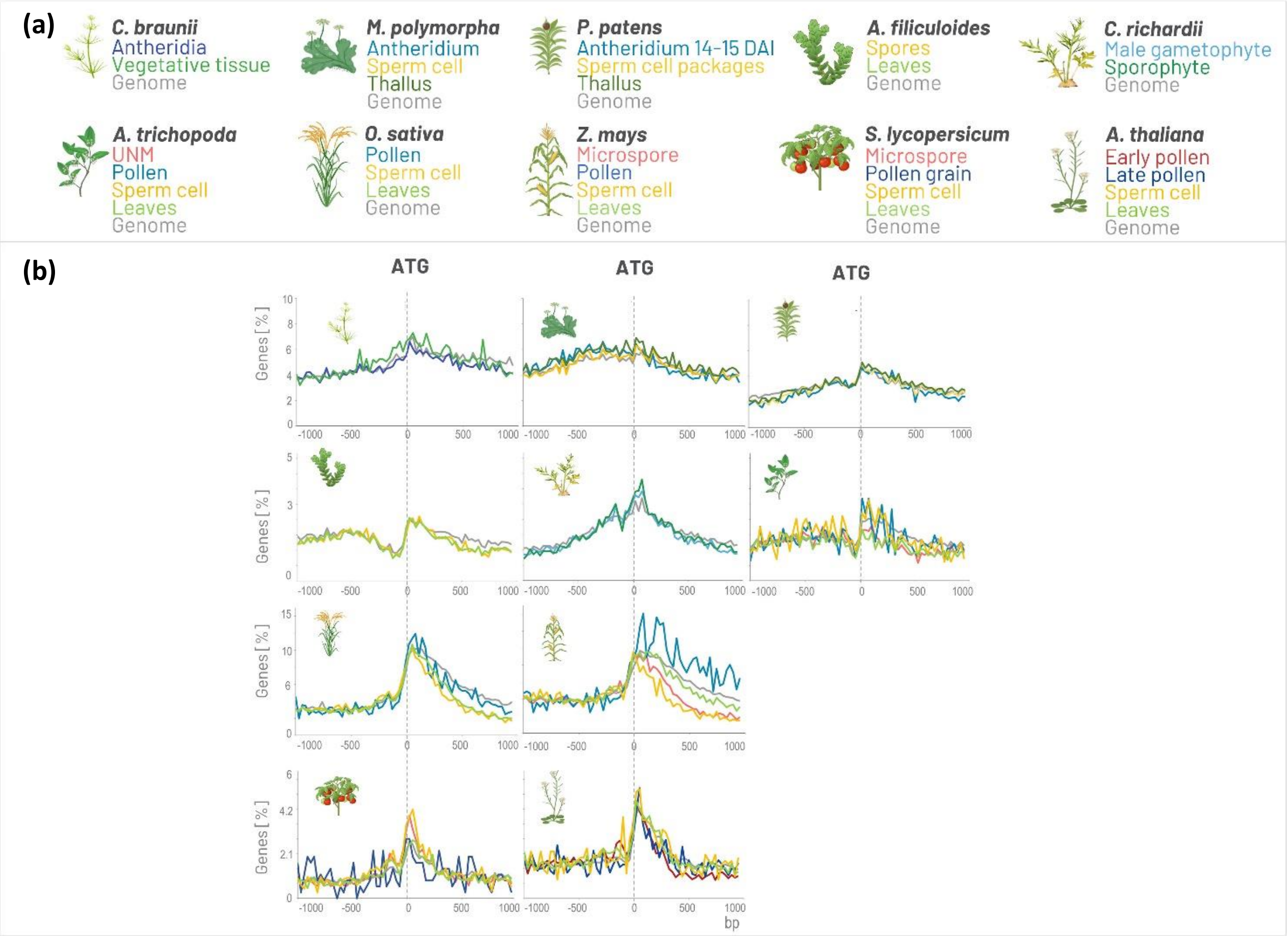

Figure S4. Example of the distribution of DRE/CRT element.

(a) The genomes analyzed across plant evolution include one streptophyte alga (*Chara*), two mosses (*Marchantia* and *Physcomitrium*), two ferns (*Azolla* and *Ceratopteris*), two monocots (*Oryza* and *Zea*), and two dicots (*Solanum* and *Arabidopsis*), as well as selected tissues and developmental stages.

(b) The distribution of DRE/CRT (CCGAC) element shows a gravitating around the start codon with higher occurrence in gene body than in 5' UTR region. This element seems to be over-represented in monocot genes. The genes expressed in the 90th percentile are shown within the range <-1000, 1000> bp, with a bucket size 30 bp; bp, base pair. The axis size was adjusted in each row.
